# Psychiatric Features of Children with Chronic Functional Constipation: Focusing on Individuals with Autism Spectrum Disorder

**DOI:** 10.1007/s10803-023-06228-8

**Published:** 2024-01-24

**Authors:** Fumiaki Akama, Katsunaka Mikami, Yasushi Orihashi, Syunya Takase, Kyuta Hanawa, Keita Nishikawa, Natsuru Watanabe, Keitaro Kimoto, Yuki Takahashi, Yuichi Onishi, Juan Salas, Kenji Yamamoto, Shigeru Ueno

**Affiliations:** 1 Department of Psychiatry, Tokai University School of Medicine, 143 Shimokasuya, Isehara, Kanagawa 259-1193 Japan; 2 Division of Clinical Research, Kitasato University Hospital, 1-15-1 Kitasato, Minami-ku, Sagamihara, Kanagawa 252-0375 Japan; 3 Cancer and Blood Disease Institute, Division of Oncology, Cincinnati Children’s Hospital Medical Center, 3333 Burnet Ave ML2011, Cincinnati, OH 45229 USA; 4 Pediatric Surgery, Tokai University, Tokai University School of Medicine, Tokyo, Japan; 5 Division of General Medicine, Okamura Isshindow Hospital, 1-7, 2-chome, Saidaiji-Minami, Okayama City, 704-8117 Okayama Japan

**Keywords:** Chronic Functional Constipation, Autism Spectrum Disorder, Abnormal Behavior, Infants, Children

## Abstract

Purpose: The present study aimed to assess the psychiatric characteristics of children with chronic functional constipation using the Aberrant Behavior Checklist-Japanese version and the Pervasive Developmental Disorders/Autism Society Japan Rating Scale, and to examine the frequency of autism spectrum disorder in children with chronic functional constipation. We also investigated differences in treatment duration between children with and without autism spectrum disorder. Methods: Treatment outcomes were examined retrospectively for 55 participants (chronic functional constipation group: n = 30, mean age 3.4 years; control group: n = 25, mean age, 4.5 years). The association between chronic functional constipation and autism spectrum disorder was evaluated using multivariable logistic regression analysis. Results: The mean Aberrant Behavior Checklist score and frequency of individuals with autism spectrum disorder were significantly higher in the chronic functional constipation group. After adjusting for age and sex, chronic functional constipation was significantly associated with autism spectrum disorder. In the chronic functional constipation group, the frequency of onset was significantly higher in children with autism spectrum disorder under 1 year of age. When treated, the mean duration of constipation was significantly longer in children with autism spectrum disorder. Conclusion: Pediatricians, pediatric surgeons, and child psychiatrists should work closely to ensure appropriate treatment of chronic functional constipation in children with autism spectrum disorder.

Constipation is one of the most prevalent gastrointestinal disorders in infants and children, with a substantial proportion of children suffering from functional constipation (Loening-Baucke, 2005). Chronic functional constipation (CFC) affects 0.7–29.6% of children and is often difficult to treat (Mugie et al., 2011; van den Berg et al., 2006) because these children often have dietary problems such as irregular eating habits, they are often reluctant to take medicine, or are afraid of treatment. In addition, CFC may lead kids to lose their urge to defecate, making it harder for them to develop regular toilet habits (Tunnessen, 1999). CFC is often associated with infrequent and/or painful defecation, fecal incontinence, and abdominal pain which causes significant distress to the child and family (Tabbers et al., 2014). A recent review identified growing evidence that CFC severely impairs health-related quality of life in patients and their families, as well as having a considerable influence on health-care costs. (Belsey et al., 2010). Although CFC may have several etiologies, in most children presenting with the condition, no underlying medical condition responsible for the symptoms can be found (Tabbers et al., 2014).

Autism spectrum disorder (ASD) is a complex developmental condition that involves persistent challenges in social interaction, speech, nonverbal communication, and restricted/repetitive behaviors (DSM-5) (American Psychiatric Association, 2013). Medical comorbidities are common in patients with ASD including functional gastrointestinal disorders (fGIDs), which are reported in 30–70% of patients with ASD (Buie et al., 2010; Penzol et al., 2019; Valicenti-McDermott et al., 2006; Wang et al., 2011), the most common of which is constipation (Penzol et al., 2019; Wang et al., 2011). One retrospective study demonstrated that at least one fGID was present in 30.5% of 845 patients with ASD, with constipation being the most prevalent (47.4% of fGID patients) (Penzol et al., 2019). A review of 144 studies published from 1980 to 2017, including studies that assessed gastrointestinal (GI) symptoms or conditions, reported that the prevalence of constipation in patients with ASD was 4.3–45.5% (median, 22%) (Holingue et al., 2018). A meta-analysis of studies evaluating GI symptoms in children with ASD demonstrated that these children experienced significantly more general GI symptoms than their control counterparts (McElhanon et al., 2014). Children with ASD who have GI symptoms like constipation have a wide range of serious problems including irritability (Bresnahan et al., 2015), anxiety, and affective disorders (Fulceri et al., 2016; Valicenti-McDermott et al., 2006). Furthermore, a study using the Aberrant Behavior Checklist to compare behavioral problems in children with ASD, with and without GI symptoms, reported that children with ASD having GI symptoms had significantly more behavioral problems, especially social withdrawal and irritability (Chaidez et al., 2014).

Although many reports have revealed that ASD tends to cause constipation, it remains unclear whether there is a higher incidence of ASD among children with CFC. To elucidate this relationship, attention must be paid to the psychiatric and developmental characteristics of children with CFC. However, few studies have been carried out on this particular subject. First, we hypothesized that children with CFC would have a higher frequency of psychiatric and developmental characteristics; therefore, the primary aim of the present study was to evaluate the psychiatric characteristics of children diagnosed with CFC and to examine the frequency of ASD in children with CFC. Next, we hypothesized that CFC in children with ASD would be more difficult to treat. Therefore, our subsequent investigation focused on differences in treatment duration between children with and without ASD.

## Methods

### Participants

The present report consisted of two studies, a cross-sectional and a follow-up study. The cross-sectional study included children aged 2–15 years with CFC who visited the Department of Pediatric Surgery at our institution between July 2013 and March 2019. Patients with organic constipation due to Hirschsprung disease, aproctia, schistorrhachia, hypothyroidism, and drug-induced constipation (psychotropic medication, etc.) were excluded from the study. Patients aged 2–15 years without constipation who were examined at the Department of Pediatric Surgery at our institution were assigned to the control group. While we intended to obtain written consent from children aged ≥ 12 years, the maximum age of the participants was 10 years; therefore, written consent was obtained from guardians at the time of the cross-sectional study. The first cross-sectional study was approved by the Institutional Review Board for Clinical Research at Tokai University School of Medicine (ref, # 13R-106).

In the present study, children in the CFC group were enrolled consecutively, and all were treated for constipation following the guidelines for CFC treatment (Tabbers et al., 2014) until remission. The outcomes of the treatment for and the duration of CFC through February 2021 were retrospectively examined. The duration of CFC was defined as the time from the onset of constipation to the end of treatment. The next follow-up study was also approved by the Institutional Review Board for Clinical Research at Tokai University School of Medicine (ref, # 20R-394).

### Measures

Chronic functional and organic constipation were diagnosed by a pediatric surgeon based on the Rome III criteria (Hyman et al., 2006; Rasquin et al., 2006). ASD was diagnosed by a child psychiatrist using the Pervasive Developmental Disorders Autism Society Japan Rating Scale (PARS) (Ito et al., 2012) for both infantile and current ASD symptoms. The PARS is a semi-structured interview that assesses autistic symptoms and behaviors as defined in the Diagnostic and Statistical Manual (fourth edition), text revision (DSM-IV-TR) (American Psychiatric Association, 2000) based on information provided by caregivers. The PARS infantile rating includes four subscales: social communication, sensitivity/difficulty, stereotyped behavior, and restricted interest (Ito et al., 2012). ASD is suspected when the PARS score is ≥ 9 in preschoolers or ≥ 13 in primary school children (Ito et al., 2012). The evaluation of each item in the PARS, the validity of which has already been established, is based on a numerical rating ranging from 0 to 2, with higher scores indicating more severe autistic symptoms (Ito et al., 2012). A diagnosis of ASD was confirmed by child psychiatrists using the DSM-5 criteria (American Psychiatric Association, 2013).

The Child Behavior Checklist (CBCL) and Aberrant Behavior Checklist-Japanese version (ABC-J) were used to evaluate the psychiatric characteristics of children with CFC based on the responses provided by their caregivers. The CBCL (Achenbach, 1991, 1992; Achenbach et al., 1987) and ABC-J (Aman et al., 1985, 2006) were both designed to provide standardized reports of recent emotional and behavioral problems. In the CBCL, problematic behaviors were scored in terms of eight characteristics (withdrawal, somatic complaints, anxiety/depression, social problems, thought problems, attention problems, delinquent behavior, and aggressive behavior) and three scales (internalizing, externalizing, and total problems) (Achenbach, 1991, 1992; Achenbach et al., 1987). The score ranges from 0 to 100, with lower scores indicating higher functioning children, while a CBCL score ≥ 64 is classified as abnormal (Achenbach, 1991, 1992; Achenbach et al., 1987). The ABC-J is a 58-item behavioral rating scale used to measure behavioral problems across five subscales (irritability and agitation, lethargy and social withdrawal, stereotypical behavior, hyperactivity and noncompliance, and inappropriate speech), in which higher scores indicate more severe behavioral problems (Aman et al., 1985). The Japanese version of the Aberrant Behavior Checklist-Community (ABC-Community) (Aman et al., 2006) had essentially the same factor structure of the original developed by Aman et al. (1985). The factor validity and reliability of ABC-Community was assessed by rating 322 Japanese subjects with moderate to profound mental retardation (Ono, 1996). Furthermore, the ABC-J is a useful tool for evaluating problematic behaviors in individuals with ASD (Kaat et al., 2014), and the Japanese versions of the CBCL (Itani et al., 2001; Nakada et al., 1999) and ABC-J (Karabekiroglu & Aman, 2009; Ono, 1996) have good reliability and validity for infants and children.

### Statistical Analyses

Continuous variables are presented as mean ± standard deviation and were compared using the Mann − Whitney *U* test. Categorical variables are presented as frequencies and percentages and were compared using Fisher’s exact test for binary variables and the Mann − Whitney *U* test for ordered variables. The association between CFC and ASD was evaluated using multivariable logistic regression analysis.

## Results

In the first cross-sectional study, the CFC group was selected from a consecutive sample of 34 patients of whom four patients refused to participate. Finally, the CFC group comprised 30 patients (18 boys, 12 girls; mean age, 3.4 years) and the control group had 25 participants (15 boys, 10 girls; mean age, 4.5 years). The age of the participants ranged from 2 to 10 years (Table 1).

**Table 1:** Characteristics of the CFC and non-CFC groups

	CFC	Non-CFC	*P*-value
	n = 30	n = 25	
Age, mean (SD)	3.4 (1.4)	4.5 (2.2)	0.0709
Boys, number (%)	18 (60.0)	15 (60.0)	1.0000
Individuals with ASD, number (%)	22 (73.3)	3 (12.0)	< 0.0001
Mean total score of CBCL (SD)	28.6 (18.8)	18.7 (15.1)	0.0506
Mean total score of ABC-J (SD)	13.4 (18.3)	4.0 (5.8)	0.0298

ABC-J, Aberrant Behavior Checklist-Japanese version; ASD, autism spectrum disorder; CBCL, Child Behavior Checklist; CFC, chronic functional constipation; SD, standard deviation

The original study was designed for children aged 2–15 years old, but the actual participants were up to 10 years of age. In the CFC group, there were no other physical diseases that induced constipation and no history of psychiatric hospital visits. In the control group, 18 participants were diagnosed with inguinal hernias; two with testicular masses; and one each with persistent urachus, bleeding hemorrhoids, preauricular subcutaneous tumor, a subcutaneous tumor on the back, and umbilical hernia.

The mean total ABC-J score was significantly higher in the CFC than in the control group, while the CBCL score was only marginally higher (*P* = 0.0506) (Table 1). Additionally, the frequency of ASD was significantly higher in the CFC than in the control group (Table 1). According to the PARS subitems, participants in the CFC group experienced more extreme faddiness than those in the control group (*P* = 0.0354). The ABC-J subscales (a) irritability and agitation and (b) lethargy and social withdrawal showed significantly higher scores in the CFC than the control group (Table 2), while hyperactivity and noncompliance showed marginally higher scores (Table 2). Since there was no significant difference in the total CBCL scores between the groups, subscale items were not examined. After adjusting for age and sex, CFC in children was significantly associated with ASD (*P* = 0.0001, using logistic regression).

**Table 2:** Mean score of the subscales of ABC-J for individuals with and without CFC

	CFC	Non-CFC	*P*-value
	n = 22	n = 21	
Mean subscale scores of ABC-J (SD)			
Irritability and agitation	4.8 (6.2)	1.4 (2.2)	0.0213
Lethargy and social withdrawal	2.5 (4.1)	0.7 (2.4)	0.0057
Stereotypic behavior	0.2 (0.6)	0.0 (0.0)	0.1722
Hyperactivity and noncompliance	5.1 (8.2)	1.7 (2.9)	0.0610
Inappropriate speech	0.9 (1.8)	0.4 (0.8)	0.2741

ABC-J, Aberrant Behavior Checklist-Japanese version; ASD, autism spectrum disorder; CFC, chronic functional constipation; SD, standard deviation

In the follow-up study, when evaluating for treatment outcomes of the 30 children with CFC, 24 children achieved remission, while the remaining six children continued to have constipation. All six children with persistent constipation had ASD, and the mean duration of CFC was significantly longer in children with ASD (Table 3). Additionally, the frequency of CFC onset at < 1 year of age was significantly higher in children with ASD than in those without (Table 3).

**Table 3:** The frequency of CFC onset and duration for children with and without ASD

	ASD	Non-ASD	*P*-value
The frequency of onset for CFC under 1 year of age (N)	11	1	0.0492
Over 1 year of age (N)	10	8	
Duration of constipation (months), mean (SD)	69.8 (29.8)	44.9 (19.6)	0.0298

ASD, autism spectrum disorder; CFC, chronic functional constipation; N, number; SD, standard deviation

## Discussion

To the best of our knowledge, the present study is the first that aimed to elucidate the psychiatric features of children with CFC. Children with CFC exhibited abnormal and problematic behaviors, such as irritability, agitation, lethargy, and social withdrawal. Compared to children without CFC, those with CFC had a higher incidence rate of ASD. In children with CFC who were diagnosed with ASD, the onset of constipation was earlier and more frequent within 1 year of birth, and the duration of CFC was longer than that in children without ASD.

It is documented that children with ASD have a higher frequency of constipation (Bresnahan et al., 2015); however, in the present study, we elucidated that children with CFC, had a higher prevalence of ASD and that the duration of CFC was longer in children with ASD. The majority of cases of childhood constipation are due to CFC and the etiology is unknown, but when we evaluated children with CFC, we found that the most of them (73.3%) had ASD. This is a new finding and may lead to the elucidation of the cause of CFC. In the past, psychiatrists encountering children with ASD considered GI disorders, especially constipation, because GI symptoms affect the core symptoms of ASD (Buie et al., 2010). In contrast, the present study suggests that pediatricians and pediatric surgeons encountering children with CFC should consider the possibility of comorbid ASD. In other words, when treating a child with CFC with drug therapy, if the child also has ASD, pediatricians and pediatric surgeons should consider coordinating treatment plans with psychiatrists. Clinicians must establish a nursing care system for children with ASD to reduce the overall burden on their families and improve environmental factors. Psychosocial influences strongly affect CFC in children (Constipation Guideline Committee of the North American Society for Pediatric Gastroenterology, Hepatology and Nutrition, 2006; Felt et al., 1999; Pashankar, 2005); therefore, treating ASD can, in turn, help treat CFC. For children with refractory CFC, in particular, clinicians must be aware that the child may have ASD.

Functional defecation disorders in ASD are associated with sensory irritability, intestinal motility, and muscle coordination problems (Peeters et al., 2013). Moreover, ASD is thought to result in slow stool transit and involves autonomic nervous system (ANS) problems (Pang & Croaker, 2011). Therefore, ASD is considered a cause of CFC based on the effects of ANS problems. Second, transitioning from bottles or breastfeeding to baby food and inappropriate toilet training may lead to constipation (Faure et al., 2017; Hyman et al., 2006). Children with ASD may also have problems changing toilet habits, (Whiteley, 2004) leading to constipation (D’Cruz et al., 2013). Third, dietary effects, such as an unbalanced diet (Eswaran et al., 2013) and genetic factors (Peeters et al., 2011), are thought to cause CFC in children. If the intake of fruits and vegetables containing dietary fiber is unbalanced, the intestinal transit time of stool is prolonged, resulting in constipation (Eswaran et al., 2013). The results of a large prospective cohort study comprising 114,516 Norwegian children indicated that children with ASD had a significantly increased odds of having constipation (adjusted odds ratio, 3.4; 95% confidence interval, 2.1–5.5) in the 6–36-month age range compared with developmentally typical children (Bresnahan et al., 2015). The results of the aforementioned study also indicated that constipation could be related to an unbalanced diet, genetic factors, immunity, metabolism, and serotonin signaling. Children with ASD tend to have constipation because they consume many unbalanced and processed foods instead of fruits and vegetables that provide dietary fiber (Eswaran et al., 2013; McElhanon et al., 2014). In the present study, we observed similar results in that children in the CFC group who were more frequently diagnosed with ASD had a more unbalanced diet.

Based on the present study’s findings, we believe that the pathophysiology of childhood CFC is strongly related to that of constipation in children with ASD. In the present study, the average total ABC-J score was significantly higher in the CFC than in the control group, indicating that the CFC group was more likely to exhibit behavioral problems. These results can be attributed to the high frequency of ASD in the CFC group. Constipation in children with ASD has been reported to be related to behavioral problems and anxiety symptoms (Chaidez et al., 2014; Fulceri et al., 2016); therefore, GI symptoms may contribute to problematic behavior in children with ASD (Wang et al., 2011). Essentially, unless the negative chain reaction due to the brain–gut interaction is disrupted, neither the problematic behavior nor the constipation will improve. If clinicians simultaneously treat constipation and problematic behaviors in children with ASD, brain–gut interactions may positively affect both. The clinicians treating children with CFC are primarily pediatricians and pediatric surgeons; therefore, the possibility of ASD as a comorbidity must always be considered when encountering CFC in children. Furthermore, when children with constipation have ASD, the treatment plan should account for both constipation and psychosocial issues, as approaching these issues simultaneously will positively impact both.

The present study had some limitations. First, this was a cross-sectional study; therefore, the co-treatment of children with CFC or ASD was not evaluated. Second, this was a single-hospital observational study; therefore, similar studies should be conducted in multiple hospitals. Third, we only evaluated whether or not children with CFC were diagnosed with ASD. Other disorders, such as depression and anxiety, must be evaluated in future studies. Fourth, PARS is based on DSM-IV criteria; therefore, we had no other option but to use PARS (DSM-IV) even though ASD is diagnosed using DSM-5 in Japan.

## Conclusion

In this study population, children with CFC had a high frequency of ASD and that children with ASD had a longer duration of CFC and experienced CFC earlier in life. If clinicians encounter children with CFC and behavioral problems, they should consider the possibility of ASD as a comorbidity. When children with CFC develop ASD, pediatricians, pediatric surgeons, and child psychiatrists should work closely together to treat both CF and ASD effectively and immediately.

## Data Availability

The datasets generated and analyzed during the current study are not publicly available due to obligation to secrecy towards the participants.
